# Intraoperative Ultrasound-Guided Conserving Surgery for Breast Cancer: No More Time for Blind Surgery

**DOI:** 10.1245/s10434-023-13900-x

**Published:** 2023-08-22

**Authors:** Massimo Ferrucci, Francesco Milardi, Daniele Passeri, Luaya Fabrizio Mpungu, Andrea Francavilla, Matteo Cagol, Tania Saibene, Silvia Michieletto, Mariacristina Toffanin, Paola Del Bianco, Ugo Grossi, Alberto Marchet

**Affiliations:** 1 Breast Surgery Unit, Veneto Institute of Oncology IOV, Istituto di Ricovero e Cura a Carattere Scientifico (IRCCS), Padua, Italy; 2 General Surgery, Department of Surgery, Oncology and Gastroenterology, University of Padua, Padua, Italy; 3 Breast Surgery Unit, Department of Surgery, Villa Salus Hospital, Venice, Italy; 4 Unit of Biostatistics, Epidemiology and Public Health, Department of CardiacThoracic Vascular Sciences and Public Health, University of Padua, Padua, Italy; 5 Clinical Trials and Biostatistics, Veneto Institute of Oncology IOV, Istituto di Ricovero e Cura a Carattere Scientifico (IRCCS), Padua, Italy

## Abstract

**Background:**

Breast-conserving surgery (BCS) still remains a blind surgery despite all available tumor localization methods. Intraoperative ultrasound (IOUS) allows real-time visualization during all resection phases.

**Methods:**

This was a prospective observational cohort study conducted at the Veneto Institute of Oncology between January 2021 and June 2022. Patients with ductal carcinoma in situ, T1-2 invasive cancer, or post-neoadjuvant tumors, suitable for BCS, were recruited. All breast cancer lesion types were included, i.e. solid palpable, solid non-palpable, non-solid non-palpable, and post-neoadjuvant treatment residual lesions. Eligible participants were randomly assigned to either IOUS or traditional surgery (TS) in a 1:1 ratio. The main outcomes were surgical margin involvement, reoperation rate, closest margin width, main specimen and cavity shaving margin volumes, excess healthy tissue removal, and calculated resection ratio (CRR).

**Results:**

Overall, 160 patients were enrolled: 80 patients were allocated to the TS group and 80 to the IOUS group. IOUS significantly reduced specimen volumes (16.8 cm^3^ [10.5–28.9] vs. 24.3 cm^3^ [15.0–41.3]; *p* = 0.015), with wider closest resection margin width (2.0 mm [1.0–4.0] vs. 1.0 mm [0.5–2.0] after TS; *p* < 0.001). Tumor volume to specimen volume ratio was significantly higher after IOUS (4.7% [2.5–9.1] vs. 2.9% [0.8–5.2]; *p* < 0.001). IOUS yielded significantly better CRR (84.5% [46–120.8] vs. 114% [81.8–193.2] after TS; *p* < 0.001), lower involved margin rate (2.5 vs. 15%; *p* = 0.009) and reduced re-excision rate (2.5 vs. 12.5%; *p* = 0.032).

**Conclusions:**

IOUS allows real-time resection margin visualization and continuous control during BCS. It showed clear superiority over TS in both oncological and surgical outcomes for all breast cancer lesion types. These results disfavor the paradigm of blind breast surgery.

Breast-conserving surgery (BCS) is the standard of care for early-stage breast cancer (BC).^1–3^ The primary goal of BCS is to ensure oncological adequacy by removing the tumor with clear resection margins, while the secondary goal is to reduce the excess of healthy tissue removal, allowing for smaller excision volumes. Nowadays, BCS could still be considered as a blind surgery since breast tumors and resection margins are not under the surgeon’s real-time direct view during all resection phases. It is palpation-guided for palpable breast lesions, and palpation may lead to imprecise excisions, as the distance from the tumor edge to the resection margin cannot be objectively measured. Resection of non-palpable BC is still a challenge for surgeons and many different techniques have been proposed to guide surgery over the years:^4^ wire-guided localization, radio-guided occult lesion localization (ROLL), radioactive seed, magnetic seed, carbon localization, and skin tattooing. Each of these techniques showed its own limitations:^5,6^ landmark displacement, poor accuracy, magnetic resonance artefacts, radiology and nuclear medicine issues, patient discomfort and extra costs. Reported rates of margin involvement after BCS range from 10 to over 40%,^6,7^ and positive resection margin is one of the strongest predictive factors for local recurrence.^8^ This event requires additional surgical procedures (re-excision or mastectomy) and/or radiotherapy boost, leading to higher healthcare costs and worse cosmetic outcomes, in addition to further stress for surgeons and patients. In 1988, Rifkin et al.^9^ first described ultrasound (US) as a tool for localizing breast masses during surgery and facilitating their surgical excision. Many other successful experiences followed, both for palpable^7,10–13^ and non-palpable^14,15^ BC. Nevertheless, the systematic use of intraoperative US during BCS is still regarded as a new developing concept and is unreasonably underused in this surgical oncology field. Intra-Operative Ultrasound-guided Surgery (IOUS) is the only technique allowing a real-time visualization of BC and continuous control of resection margins during all surgical phases. Previous series reported glaring advantages over the other techniques in terms of oncological and surgical outcomes.^7,10–15^

Our study aims to prospectively compare IOUS with traditional (palpation- or wire-guided) surgery (TS) for all types of BC lesions with regard to involved resection margins and reoperation rates, as well as volumes of breast tissue excised.

## Materials and Methods

This was a prospective observational cohort study conducted between January 2021 and June 2022 at Veneto Institute of Oncology. The protocol was approved by the local Ethics Committee and all patients provided informed consent.

### Inclusion and Exclusion Criteria

Patients diagnosed with ductal carcinoma in situ (DCIS), T1-2 invasive BC or post-neoadjuvant chemotherapy (NACT) BC residual lesion, deemed suitable for BCS, were consecutively enrolled. In order to ensure homogeneity and enable a valid comparison between the two groups, we devised a clinical-radiological categorization system for all BC lesion types in four groups: solid palpable (type A); solid non-palpable (type B); non-solid non-palpable [e.g., microcalcification clusters, architectural distortive areas] (type C); post-NACT residual lesions (type D). All these lesions were included in this study.

Patients were categorized into two groups according to their body mass index (< 30 and ≥ 30 kg/m^2^). Patients with multicentric tumors, inflammatory disease, previous ipsilateral BCS, previous ipsilateral radiotherapy, as well as all patients who were candidates for mastectomy, were excluded. Patients with a BC lesion, or its localizing metallic marker, not clearly detectable at preoperative US examination were also excluded.

### Study Characteristics

Eligible participants were randomly assigned to either IOUS or TS in a 1:1 ratio, generating two homogeneous groups. During the first 6 months of the study period, we enrolled only patients with types A and B lesions in both groups in order to get familiar with the new technique; thereafter, we started recruiting also patients with types C and D lesions. Oncological outcomes were closest margin width, surgical margin involvement, and reoperation rates. Surgical outcomes were main specimen and cavity shaving margin volumes, excess healthy tissue removal, calculated resection ratio (CRR), excision times, and postoperative complications rate. All BC non-palpable lesions assigned to TS were preoperatively localized with the traditional wire-guided technique. The same surgeon (MF), with proven experience in TS and already skilled in basic breast US, performed all the surgical procedures.

### Preoperative Work-Up

All patients underwent a comprehensive preoperative evaluation with digital mammography, ultrasonography, and a proven histologic diagnosis by core-needle biopsy or vacuum-assisted breast biopsy. Type C lesions were marked with a nitinol ring-shaped clip at the time of vacuum-assisted breast biopsy. The same US-visible tissue marker was also placed in the core of the BC in all patients starting a neoadjuvant treatment. All patients assigned to IOUS were re-evaluated in an outpatient setting a few days before surgery to confirm the US detectability of BC lesions or their corresponding metallic marker. Whenever a tissue marker was placed, an accurate tumor extent mammogram evaluation was performed to identify the required wideness of tissue removal around the clip. In our experience, this patient-tailored accurate work-up made preoperative localization device positioning not necessary in the IOUS group.

### IOUS Technique

A GE Healthcare LOGIQ S8 US machine equipped with a linear array probe (GE Healthcare ML 6-15) and a dedicated intraoperative hockey stick probe (GE Healthcare L8-18i) was used for all IOUS procedures.

**Fig. 1:**
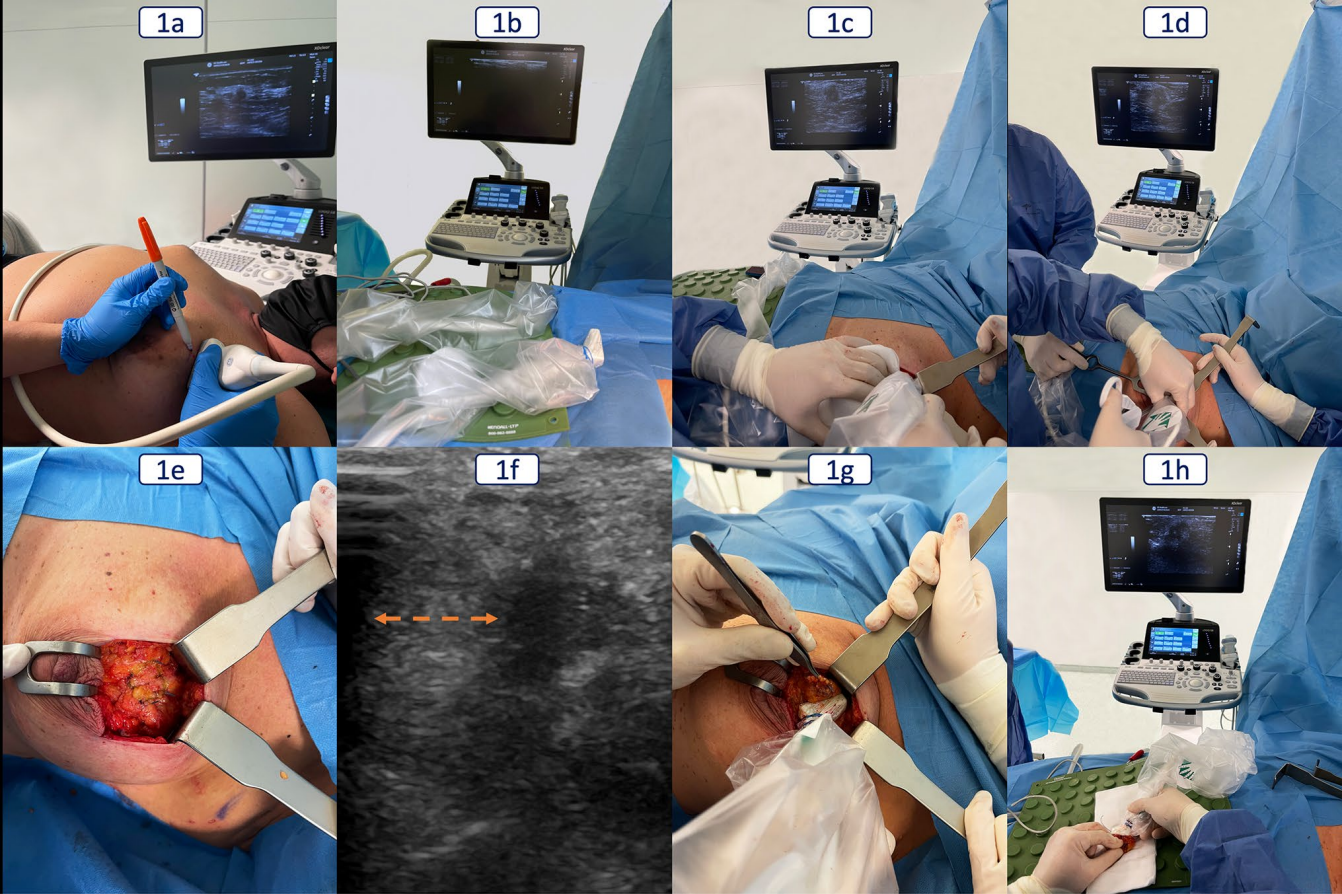
Intra-Operative Ultrasound-guided Surgery (IOUS) personal technique

On the day of surgery, in the pre-anesthesia room, the surgeon first performed an US scan of the targeted lesion with the linear probe to localize and draw a skin projection of the tumor defining the optimal skin incision site (Fig. 1a). The tumor distance from the skin was assessed to determine if skin resection was necessary. In the operating room, a sterile coverage of intraoperative hockey stick probe and an ergonomic position of US machine (in front of the surgeon) was guaranteed (Fig. 1b). After the skin incision, we achieved an optimal glandular exposure through a wide skin and subcutaneous layer undermining (Fig. 1c). The hockey stick probe allowed a precise scan of the tumor contour above the glandular surface, facilitating the definition of the superficial perimeter for a safe resection. The four cardinal points of the resection area were marked with metallic surgical clips (Fig. 1d) and then connected by a superficial electrocautery mammary gland incision (Fig. 1e). US confirmation of the safety distance between this superficial glandular incision and each tumor edge was achieved (Fig. 1f).

We performed a wide local excision (WLE) of the tumor with US real-time continuous control of all the resection margins (Fig. 1g). At the end of the excision, the ex vivo US examination (Fig. 1h) showed how the tumor was centralized inside the specimen and allowed a precise assessment of resection margin width, guiding the cavity shaving extent. A specimen x-ray was performed whenever a tissue marker was applied before surgery, to confirm its inclusion, together with any residual microcalcifications. Excision time was defined as the time (in minutes) between the skin incision and the complete removal of surgical specimens. Systematic circumferential tumor-bed resection margin cavity shaving was performed in all cases, both in the IOUS and TS groups. Associated axilla surgical treatments were reported. All patients underwent BCS associated with level I and level II glandular reshaping oncoplastic techniques, according to tumor location and tumor/breast volume ratio. The additional operating time required and healthy breast tissue removed exclusively for oncoplastic procedures were not considered in the analysis.

### Histological Characteristic and Volume Calculations

All pathological details were collected.^16,17^ The presence of an invasive tumor-associated DCIS component was reported and categorized as focal or extended. For each patient, the three dimensions of the tumor, main specimen, and cavity shaving margins were recorded. Tumor volume was calculated considering the tumor as an elliptical sphere using the ellipsoid equation *V* *=* 4/3π*a*b*c, where *a*, *b* and *c* are the lengths of the semi-diameters of the tumor. The main specimen and cavity shaving margin volumes were calculated as volumes of a rhombic prism, using the formula *V* *=* *d*1*d2*h/2, where *d*1, *d*2 and *h* are the diameters of the main specimen and of each single cavity shaving margin.

The closest margin width was calculated as the minimal distance between the tumor and the closest resection margin. The optimal resection volume (ORV) was defined as the spherical tumor volume, with *r* = the main dimension of the tumor plus an additional 1 cm clear margin. The CRR was intended as the specimen volume related to the ORV. Reoperation was recommended for any resection margin < 2 mm for DCIS^18^ and for the presence of ‘ink on tumor’ for the invasive component;^19^ involved resection margins and reoperation rates were reported. All postoperative complications and adjuvant treatments were described.

### IOUS Learning Curve

In the IOUS group, we identified three 6-month periods in which to assess a specific learning curve by analyzing the IOUS performances on main outcomes over time. During the first 6 months, we only dealt with type A and B lesions as they were the easiest to be identified by US scan. After 6 months’ experience, we gradually faced the other types (C and D), which were more challenging and required a longer learning curve to be detected.

### Statistical Analyses

Continuous variables were expressed as median ± interquartile range (IQR), and categorical variables were expressed as number (%). Continuous variables were compared using the Wilcoxon or Kruskal–Wallis rank-sum tests, while categorical variables were compared using the Chi-square or Fisher's exact tests. Single and multiple logistic regression models were built to estimate the association between the use of IOUS and two binary outcomes of interest: probability of margin involvement and need for reoperation. Covariates for the multiple logistic regression were selected based on clinical experience. The results were reported as odds ratio (OR), 95% confidence interval (CI), and *p*-value. A *p*-value < 0.05 was considered statistically significant. Statistical analyses were performed using the R system.^20^

## Results

### Patient and Tumor Characteristics

Overall, 160 patients undergoing BCS were enrolled: 80 were allocated to the TS group and 80 to the IOUS group. Patient and tumor characteristics, as well as the surgical and complementary systemic treatments, were comparable between the two groups (Table 1). All patients were White Caucasians and the mean age was 62 years (range 37–92 years). The majority of patients (83%) had a body mass index between 18 and 30 kg/m^2^. The upper outer quadrant tumor localization counted for nearly half (49%) of all cases in both groups. Most patients (59%) were diagnosed with invasive BC of no special type. Tumor subtypes were homogeneously distributed, with luminal A BC being the most represented (47%). An invasive tumor-associated DCIS component was present in 111 cases (71%); this component was described as focal in 66 (59%) specimens and extended in the remaining specimens (41%). Associated axillary surgery was performed in 140 patients, with 116 (72%) undergoing sentinel lymph node biopsy and 24 (15%) undergoing axillary clearance. Intraoperative radiotherapy using the INTRABEAM system was administered to 21 (13%) patients, with 10 in the TS group and 11 in the IOUS group. Pathological T1 was reported in 68% of tumors, while pathological N0 was reported in 85%. Stage I was the most represented. Twenty-three (14%) patients underwent NACT, with 9 (11%) in the TS group and 14 (18%) in the IOUS group, resulting in a 35% rate of pathological complete response. Following multidisciplinary discussion, all patients received tailored adjuvant therapies and/or oncological follow-up.

**Table 1:** Patient characteristics

Characteristic		Surgical approach		
	Overall [*n* = 160]	TS [*n* = 80]	IOUS [*n* = 80]	*p*-Value^a^
Age, years [median (IQR)]	62 (51, 73)	62 (51, 73)	62 (51, 72)	0.7
BMI				0.8
<18	1 (0.6)	1 (1.3)	0 (0)	
18–24	78 (49)	40 (50)	38 (48)	
25–29	54 (34)	25 (31)	29 (36)	
≥30	27 (17)	14 (18)	13 (16)	
Tumor location				0.2
UOQ	78 (49)	39 (49)	39 (49)	
UIQ	30 (19)	12 (15)	18 (22)	
LOQ	32 (20)	17 (21)	15 (19)	
LIQ	16 (10)	9 (11)	7 (9)	
Other	4 (2)	3 (4)	1 (1)	
Breast cancer detection method				0.7
Palpation	69 (43)	36 (45)	33 (41)	
Screening	91 (57)	44 (55)	47 (59)	
Microcalcifications on mammography	47 (29.4)	25 (31.2)	22 (27.5)	0.6
Microcalcifications extent, cm [median (IQR)]	1.2 (0.9, 1.5)	1.3 (1, 1.5)	1.1 (0.9, 1.4)	0.6
Final histology				>0.9
IC NST	95 (59)	47 (59)	48 (60)	
LIC	20 (13)	10 (12)	10 (12)	
DCIS	16 (10)	9 (11)	7 (9)	
Other	29 (18)	14 (18)	15 (19)	
Luminal type				0.6
Luminal A	68 (47)	35 (48)	33 (46)	
Luminal B	55 (38)	25 (34)	30 (42)	
HER2+	9 (6.5)	5 (7)	4 (6)	
Triple negative	12 (8.5)	8 (11)	4 (6)	
DCIS-associated component	111 (71)	59 (74)	52 (68)	0.4
DCIS-associated component type				0.6
Focal	66 (59)	37 (63)	29 (56)	
Extended	45 (41)	22 (37)	23 (44)	
Grading				0.2
G1	27 (18)	12 (15)	15 (20)	
G2	82 (54)	42 (53)	40 (55)	
G3	43 (28)	25 (32)	18 (25)	
Associated axilla surgery				0.7
No	20 (12.5)	10 (12)	10 (12.5)	
SNB	116 (72.5)	56 (70)	60 (75)	
AC	24 (15)	14 (18)	10 (12.5)	
IORT	21 (13)	10 (12)	11 (14)	0.8
Clinical tumor size – T (TNM) T1 T2	123 (77) 37 (23)	63 (79) 17 (21)	60 (75) 20 (25)	0.6
Pathological tumor size – T (TNM)				0.7
yT0	8 (5)	4 (5)	4 (5)	
T1	109 (68)	54 (68)	55 (69)	
T2	27 (17)	13 (16)	14 (17)	
TIS	16 (10)	9 (11)	7 (9)	
Pathological nodal status – N (TNM)				0.5
N0	136 (85)	70 (88)	66 (82)	
N1	19 (12)	8 (10)	11 (14)	
N2	4 (2.5)	1 (1.3)	3 (3.8)	
N3	1 (0.6)	1 (1.3)	0 (0)	
Staging				>0.9
0	24 (15)	13 (16)	11 (14)	
I	98 (61)	48 (60)	50 (62)	
II	33 (21)	17 (21)	16 (20)	
III	5 (3)	2 (3)	3 (4)	
Neoadjuvant chemotherapy	23 (14)	9 (11)	14 (18)	0.4
Adjuvant treatments				
Hormone therapy	123 (77)	63 (79)	60 (75)	0.6
CT ± trastuzumab	38 (24)	21 (26)	17 (21)	0.5
Radiotherapy	127 (79)	62 (78)	65 (81)	>0.9
Follow-up	7 (4.4)	3 (3.8)	4 (5.0)	>0.9

Data are expressed as *n* (%) unless otherwise specified

*TS* traditional surgery, *IOUS* intraoperative ultrasound-guided surgery, *IQR* interquartile range, *BMI* body mass index, *UOQ* upper outer quadrant, *UIQ* upper inner quadrant, *LOQ* lower outer quadrant, *LIQ* lower inner quadrant, *IC NST* invasive cancer of non-special type, *LIC* lobular infiltrating carcinoma, *DCIS* ductal carcinoma in situ, *HER2* human epidermal growth factor receptor 2, *SNB* sentinel lymph node biopsy, *AC* axillary clearance, *IORT* intraoperative radiation therapy, *CT* chemotherapy

^a^Wilcoxon rank-sum test, Fisher's exact test, Pearson's Chi-square test

### BC Lesion Types

**Fig. 2:**
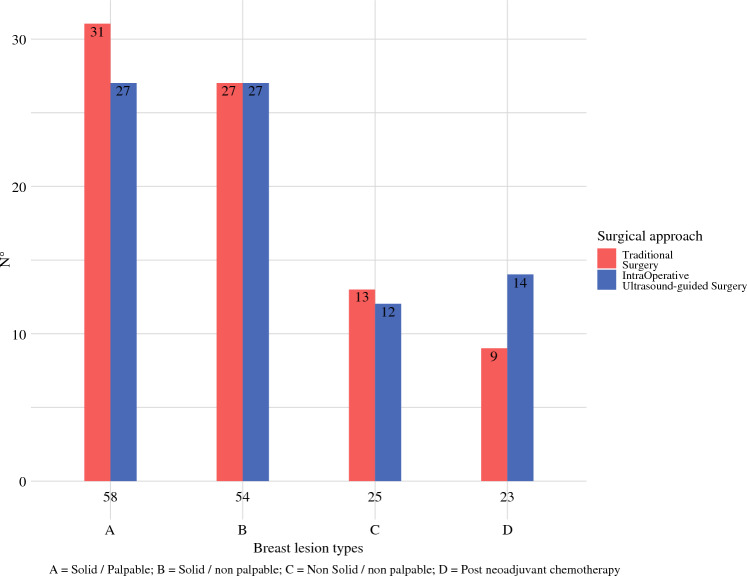
Surgical approach according to breast lesion types

The four types of BC lesions were uniformly distributed between the two treatment groups (*p* = 0.7) (Fig. 2). A total of 58 palpable BCs were enrolled: 31 (53.4%) underwent IOUS and 27 (46,6%) underwent TS. Among the 54 type B tumors, 27 (50%) were treated with IOUS and 27 (50%) were treated with TS. There were 25 type C lesions, with 12 (48%) allocated to IOUS group and 13 (52%) to the TS group. IOUS was performed in 14 patients (61%) after neoadjuvant treatments.

### Surgical and Oncological Outcomes

Surgical and oncological outcomes, according to the surgical approach, are shown in Table 2. IOUS always guaranteed an accurate localization of the targeted lesion and WLE was successfully completed via US guidance in all cases (100% sensitivity).

**Table 2:** Surgical and oncological outcomes

Characteristic		Surgical approach		
	Overall [*N* = 160]	TS [*n* = 80]	IOUS [*n* = 80]	*p*-Value^a^
Preoperative radiological main dimension, cm	1.3 (0.9, 2.0)	1.2 (0.8, 2.0)	1.3 (1.0, 1.8)	0.6
Excision time, min	24.5 (18.0, 30.0)	21.0 (16.5, 27.0)	28.0 (23.2, 31.5)	**< 0.001**
Tumor pathological main dimension, cm	1.4 (1.0, 1.9)	1.4 (0.9, 1.9)	1.5 (1.1, 1.9)	0.11
Tumor volume, cm^3^	0.8 (0.3, 1.9)	0.7 (0.2, 1.8)	0.8 (0.4, 2.2)	0.13
Main specimen volume, cm^3^	22.0 (12.6, 33.9)	24.3 (15.0, 41.3)	16.8 (10.5, 28.9)	**0.015**
Cavity shaving volume, cm^3^	5.6 (3.0, 8.8)	6.7 (3.0, 11.5)	5.3 (3.0, 7.8)	0.079
Main specimen + cavity shaving volume, cm^3^	27.5 (17.9, 42.8)	31.5 (20.3, 49.3)	25.2 (15.6, 36.8)	**0.013**
Tumor volume / main specimen volume, %	3.7 (1.6, 7.6)	2.9 (0.8, 5.2)	4.7 (2.5, 9.1)	**< 0.001**
Tumor volume / main specimen + cavity shaving volume, %	3.1 (1.3, 5.8)	2.2 (0.6, 4.2)	3.7 (1.8, 7.0)	**0.002**
ORV	20.6 (14.1, 31.1)	20.6 (12.8, 31.1)	22.4 (15.6, 31.7)	0.12
CRR, main specimen volume / ORV [%]	99.0 (60.8, 141.0)	114 (81.8, 193.2)	84.5 (46.0, 120.8)	**<0.001**
Closest margin width, mm	2.0 (1.0, 3.0)	1.0 (0.5, 2.0)	2.0 (1.0, 4.0)	**< 0.001**
Involved margins	14 (8.8)	12 (15)	2 (2.5)	**0.009**
Type of margin involvement				> 0.9
DCIS	12 (86)	10 (83)	2 (100)	
Focal involvement	3 (25)	2 (20)	1 (50)	
IC NST	1 (7.1)	1 (8.3)	0 (0)	
LIC	1 (7.1)	1 (8.3)	0 (0)	
Additional treatments				**< 0.001**
RT boost	2 (14)	2 (17)	0 (0)	
Reoperations	12 (86)	10 (83)	2 (100)	
Type of reoperation				> 0.9
WLE	10 (83)	8 (80)	2 (100)	
Mastectomy	2 (17)	2 (20)	0 (0)	
Postoperative complications	28 (18)	16 (20)	12 (15)	0.4
Type of complication				0.4
Seroma	19 (68)	10 (63)	9 (75)	
Hematoma	2 (7)	1 (6)	1 (8.3)	
Surgical site infection	6 (21)	4 (25)	2 (16.7)	
Wound dehiscence	1 (4)	1 (6)	0 (0)	

Significative *p* values (< 0.05) are reported in bold

Data are expressed as median (IQR) or *n* (%)

*ORV* optimal resection volume, *WLE* wide local excision, *IC NST* invasive cancer of non-special type, *LIC* lobular infiltrating carcinoma, *DCIS* ductal carcinoma in situ, *CRR* calculated resection rate, *TS* traditional surgery, *IOUS* intraoperative ultrasound-guided surgery, *IQR* interquartile range

^a^Wilcoxon rank-sum test, Pearson's Chi-square test, Fisher's exact test

**Fig. 3:**
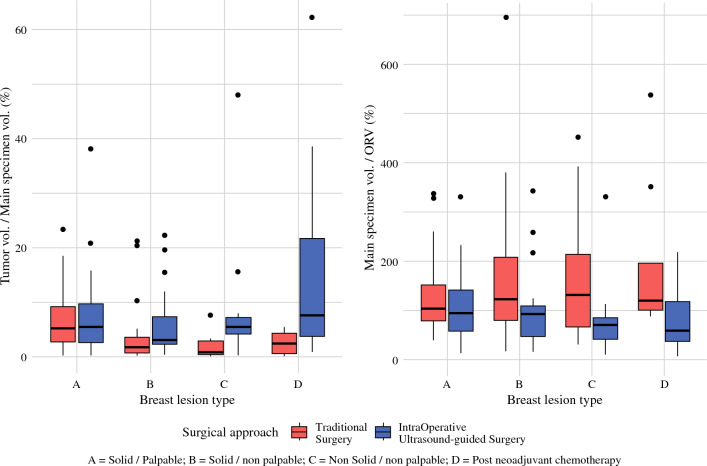
Relation between breast lesion types and surgical outcomes

Median excision time was 7 min shorter with TS (21.0 [16.5–27.0] vs. 28.0 [23.2–31.5] min with IOUS; *p* < 0.001). The main tumor dimension medians (1.4 cm [0.9–1.9] in TS vs. 1.5 cm [1.1–1.9] in IOUS; *p* = 0,11) and tumor volumes (0.7 cm^3^ [0.2–1.8] in TS vs. 0.8 cm^3^ [0.4–2.2] in IOUS; *p* = 0,11) were similar. IOUS significantly reduced both the main specimen volumes (16.8 cm^3^ [10.5–28.9] vs. 24.3 cm^3^ [15.0–41.3]; *p* = 0.015) and global main specimen plus cavity shaving margin volumes (25.2 cm^3^ [15.6–36.8] vs. 31.5 cm^3^ [20.3–49.3]; *p* = 0.013). Cavity shaving volumes were smaller in IOUS, with a borderline statistical significance (5.3 [3.0–7.8] vs. 6.7 [3.0–11.5] cm^3^; *p* = 0.079). The tumor volume to specimen volume ratio was significantly higher after IOUS (4.7% [2.5–9.1] vs. 2.9% [0.8–5.2] after TS; *p* < 0.001). Similarly, the tumor volume to specimen plus cavity shaving margin volumes ratio was significantly higher after IOUS (3.7% [1.8–7.0] vs. 2.2% [0.6–4.2] after TS; *p* = 0.002). This beneficial effect of IOUS was not greatly appreciable for palpable tumors, yet highly relevant for the other three types of BC lesions: type B (1.7 [0.6–3.5] vs. 3.0 [2.3–7.3]; *p* = 0,03), type C (0.8 [0.4–2.9] vs. 5.4 [4.1–7.2]; *p* = 0.03), and type D (2.3 [0.5–4.2] vs. 7.6 [3.8–21.7]; *p* = 0.07) [Fig. 3].

Reporting similar ORVs (*p* = 0.12), IOUS achieved significantly better CRR (84.5% [46–120.8] vs. 114% [81.8–193.2] after TS; *p* < 0.001) and wider closest margin width (2.0 [1.0–4.0] vs. 1.0 [0.5–2.0] mm; *p* < 0.001).

CRRs were consistently lower and less skewed for each type of BC lesion in the IOUS group compared with the TS group, mainly for type C and D lesions (Fig. 3). In the TS group, we observed significantly higher median global main specimen plus cavity shaving volumes in obese patients (30 cm^3^ [19–41] vs. 56 cm^3^ [33–90]; *p* < 0.001), while this difference was not observed in the IOUS group (25 cm^3^ [16–37] vs. 31 cm^3^ [18–35]; *p* = 0.5).

The involved resection margin rate was significantly higher in the TS group (15% vs. 2.5% after IOUS; *p* = 0.009). The only two cases of involved margins in the IOUS group were due to an unexpected finding of a DCIS component (100%); among the 12 cases with involved margins in the TS group, 10 (83%) were due to a DCIS component (100%) and the remaining 2 (17%) were due to an invasive component. Two TS patients with focal DCIS margin involvement received a radiotherapy boost treatment only following multidisciplinary discussion. All remaining patients with involved margins underwent reoperation, with a significantly higher rate in the TS group (12.5 vs. 2.5% after IOUS; *p* = 0.032). Mastectomy was needed for only two patients in the TS group (17%), while all remaining patients underwent surgical margin clearance.

In the binary logistic regression analysis, IOUS was found to be a protective factor over TS for both involved margins and the need for reoperations (OR 0.15 [0.02–0.56] and 0.18 [0.02–0.76]; *p* = 0.01 and 0.04, respectively), as shown in Table 3. After adjusting the analysis for neoplasm staging, BC lesion types and final histology, the protective effect of IOUS was confirmed (OR 0.12 [0.02–0.52] and 0.028 [0.02–0.66]; *p* = 0.01 and 0.03, respectively). Postoperative complication rates were similar in the two groups (20% after TS vs. 16% after IOUS; *p* = 0.4) and the most frequent type was seroma (68%); all patients were conservatively treated in an outpatient setting.

**Table 3:** Oncological outcomes according to surgical approach

Surgical approach	Involved margins			Need for reoperation		
	OR	95% CI	*p*-Value	OR	95% CI	*p*-Value
*Unadjusted*						
TS	–	–		–	–	
IOUS	0.15	0.02–0.56	**0.01**	0.18	0.02–0.76	**0.04**
*Adjusted*^*a*^						
TS	–	–		–	–	
IOUS	0.12	0.02–0.52	**0.01**	0.028	0.02–0.66	**0.03**

Significative *p* values (< 0.05) are reported in bold

*OR* odds ratio, *CI* confidence interval, *TS* traditional surgery, *IOUS* intra-operative ultrasound-guided surgery

^a^Analysis adjusted for staging, type of lesion, and final histology

### Learning Curve

**Fig. 4:**
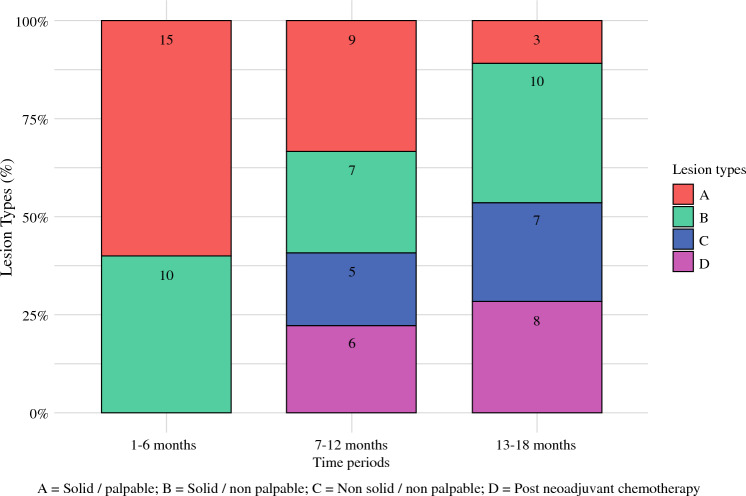
Breast lesion types distribution within IOUS group across the fixed time periods

During the three 6-month fixed periods, IOUS was performed on 25, 27, and 28 patients, respectively. The distribution of different types of BC lesions across these periods is illustrated in Fig. 4. The primary outcomes for the IOUS group, stratified by the three study periods, are presented in Table 4. There were no significant differences in the main tumor dimensions and volumes, while median excision times slightly decreased (29.7 to 28.0 to 26.2 min) across the three periods. The main specimen and cavity shaving volumes were significantly reduced between the first and last periods (21.6 vs. 11.8 cm^3^ and 5.6 vs. 3.3 cm^3^, respectively) while we observed a small increase in volumes during the second period (25.3 cm^3^ and 6.4 cm^3^, respectively). Similarly, there was an improving trend in the tumor volume to main specimen volume ratio between the first and last periods (4.3 vs. 6.0%), with a slight reduction during the second period (4.1%). Given similar ORVs (*p* = 0.8), we observed a significantly improved CRR in the last period compared with the first period (52.0 vs. 96.2%), despite worsening during the second period (107.7%). The minimal distance to the resection margin was slightly wider in the last period (2.5 vs. 2.0 mm). Since we reported only two patients with involved margins requiring reoperation in the IOUS group, no difference could be observed between the periods (Table 4).

**Table 4:** Learning curve for main outcomes within the IOUS group across the fixed time periods

	Experience time			
	A (31%) 1–6 months [*n* = 25]	B (34%) 7–12 months [*n* = 27]	C (35%) 13–18 months [*n* = 28]	*p*-Value^a^
Excision time, min	29.7 (28.0, 31.5)	28.0 (22.8, 31.5)	26.2 (20.8, 31.9)	0.2
Tumor pathological main dimension, cm	1.5 (1.1, 1.6)	1.5 (1.1, 2.2)	1.4 (1.0, 2.0)	0.8
Tumor volume, cm^3^	0.8 (0.5, 1.8)	0.9 (0.4, 2.8)	0.7 (0.3, 2.2)	0.7
Main specimen volume, cm^3^	21.6 (12.5, 32.4)	25.3 (16.0, 30.8)	11.8 (8.7, 21.7)	**0.014**
Cavity shaving volume, cm^3^	5.6 (2.8, 7.6)	6.4 (3.7, 8.6)	3.3 (2.3, 5.8)	**0.012**
Main specimen + cavity shaving volume, cm^3^	26.4 (16.2, 41.1)	31.5 (22.6, 44.3)	16.6 (11.7, 28.3)	**0.011**
Tumor volume/main specimen volume, %	4.3 (2.5, 6.6)	4.1 (2.0, 10.3)	6.0 (2.9, 12.1)	0.6
Tumor volume/main specimen + cavity shaving volume, %	3.6 (1.9, 5.8)	3.6 (1.5, 7.2)	4.4 (2.1, 7.4)	0.7
ORV	22.4 (15.6, 24.4)	22.4 (15.6, 40.2)	20.6 (14.1, 33.0)	0.8
CRR, main specimen volume/ORV [%]	96.2 (67.7, 125.3)	107.7 (57.6, 127.1)	52.0 (36.5, 80.3)	**0.013**
Closest margin width, mm	2.0 (1.0, 3.0)	2.0 (1.0, 3.5)	2.5 (1.8, 4.2)	0.2
Involved margins	1 (4.0)	1 (3.7)	0 (0)	0.5
Reoperations	1 (4.0)	1 (3.7)	0 (0)	0.5

Significative *p* values (< 0.05) are reported in bold

Data are expressed as median (IQR) or *n* (%)

*IOUS* intra-operative ultrasound-guided surgery, *ORV* optimal resection volume, *CRR* calculated resection ratio, *IQR* interquartile range

^a^Kruskal–Wallis rank sum test, Fisher's exact test

## Discussion

IOUS is a safe, cost-saving, time-efficient, non-radioactive, painless, surgeon-delivered technique, that has proven to be helpful in all BCS phases without any procedure-related complications. The IOUS detection rate was 100% for all BC lesion types, as also reported in other series.^15,21,22^ Higher tumor to specimen volume ratios, as observed in IOUS, reduced the extent of healthy tissue removal. The real-time continuous control of tumor and resection margins under direct-view guidance resulted in a significantly improved centralization of tumors within the specimen in the IOUS group. This was proved by enhanced CRR, wider closest margin width, and lower involved margins rates (Table 2).

To the best of our knowledge, this is the first study to comprehensively analyze the IOUS technique from multiple perspectives. We systematically categorized all BC lesion types in four groups and successfully employed IOUS for each category. The same surgeon (MF), with proven experience in conventional breast surgery, performed all the procedures to minimize any potential bias arising from variations in surgical experience and skills (US is strongly operator-dependent). We calculated each volume by all three spatial specimen dimensions and first described the volumes of every single cavity shaving margin, which affects the total excision volumes as much as the main specimen volumes. We also analyzed the IOUS performance in obese patients, proving its potential advantages in reducing the excess of healthy tissue removal, even within this particular patient cohort.

### IOUS Versus Palpation-Guided BCS

IOUS guarantees an objectively measured control of resection margins during BCS for palpable tumors, whereas palpation-guided surgery relies on subjective approximation of the desired distance from the tumor. We observed a lower rate of involved margins in the IOUS group compared with the TS group for palpable lesions (7.4 vs. 12.9%; *p* = 0.67), although this difference was not significant as the only two cases of positive margins reported in the IOUS group occurred in palpable lesions at the beginning of the experience.

The COBALT trial demonstrated a significant reduction in involved margin rates for palpable tumors with IOUS compared with TS (3 vs. 17%; *p* = 0.009).^6^ Similarly, a retrospective review^7^ reported a significantly lower likelihood of margin involvement with IOUS compared with TS (9 vs. 41%; *p* = 0.01) and a lower re-excision rate (9 vs. 34%; *p* = 0,04). Eggeman et al.^10^ also experienced a significant reduction in reoperation rates for positive margins with IOUS compared with TS (11.6 vs. 24.1%; *p* = 0.004).

### IOUS Versus Wire-Guided Surgery and ROLL

Esgueva et al.^14^ reported smaller CRRs in the IOUS group than in the wire-guided surgery (WGS) group (1.88 vs. 2.56; *p* = 0,006) and described a significant reduction in positive margin rates after IOUS (5.4 vs. 15.15% with WGS; *p* = 0,02), which is consistent with our findings (2.5% with IOUS vs. 15% with TS; *p* = 0.009). The same authors^14^ also reported three times higher mastectomy rates in the WGS group (2.7 vs. 9.09%; *p* = 0.04). In our study, no mastectomy was required after IOUS, unlike TS (2.5%). A randomized controlled trial conducted in Germany^23^ reported a 93 % clear margin rate after BCS in the IOUS arm compared with 65 % in the WGS control arm (*p* = 0.026).

IOUS demonstrated benefits even when compared with the ROLL technique, as described in a retrospective study^24^ reporting significantly lower rates of involved margins in the IOUS group (3.7%) compared with WGS (21.3%) and ROLL (25%) (*p* = 0.023).

### IOUS for Post-neoadjuvant BC Residual Lesions

Rubio et al.^25^ reported lower excision volumes in the IOUS group compared with WGS (34.86 vs. 49.51 mm^3^; *p* = 0.03) in patients with pathologic complete response or microscopic residual disease. In our experience with type D lesions, we observed smaller excision volumes (23.7 mm^3^ after IOUS vs. 30.4 mm^3^ after TS; *p* = 0.101) and a 0% re-excision rate in the IOUS group.

### Excision Time and Financial Implications

The median IOUS excision time was only 7 min longer than TS. The COBALT trial^6^ reported similar excision times between IOUS and TS, describing an additional 5-min operative time needed for IOUS before and after the procedure. In some cases, IOUS can also be used to confirm the inclusion of lesions and clips inside the specimen, leading to a reduction in global operative time and improved logistics.^14^

Although there is a lack of cost-efficiency studies, some authors found that the average cost of IOUS is lower than WGS, saving around $900–$925^14,26^ per surgery. A dedicated cost-benefit analysis^27^ identified a €154 cost difference between palpation-guided surgery and IOUS, which was not significant but could become relevant and cost-saving for high-volume centers, particularly when considering the reduced expense associated with decreased re-excision rates.

### IOUS Oncoplastic Surgery, Cosmetic Considerations, and Learning Curves

IOUS is particularly helpful when combined with oncoplastic procedures, since the reduction in the involved margins rate is even more important to prevent complex reoperations, as described by Barellini et al.^28^ Excised volume has been proved to be a reliable indicator of cosmetic outcomes.^29,30^ The COBALT trial^13^ demonstrated that smaller excision volumes were associated with improved overall cosmetic outcome at 1-year follow-up. Based on our experience, IOUS consistently yielded reduced overall main specimen and cavity shaving margin excised volumes for all BC lesion types. This significant finding holds promising implications for enhancing overall cosmetic outcomes.

The IOUS technique has not been widely adopted as a standard procedure in the majority of BC centers, despite multiple studies demonstrating its advantages over wire- or palpation-guided surgery. The reasons for this may include a lack of specialized training programs and the requirement of a specific learning curve, which is likely longer than that required for other localization techniques.

The optimal number of procedures required to master the IOUS technique is still debated and, to date, only a few limited observational studies have examined this crucial aspect. Esgueva et al.^14^ proposed that a minimum of 11 cases constitutes an adequate learning curve, intended as the minimum number of performed cases necessary to achieve a safe tumor excision. However, our study suggested that given basic breast US knowledge, a longer learning curve may be needed to master the technique for all types of BC lesions. Our learning curve can be divided into three distinct 6-month phases: initial proficiency development (comprising the initial 25 patients), complete skills acquisition (encompassing the subsequent 27 patients), and ultimate mastery of the technique (consisting of the final 28 patients). We felt confident to start dealing with more challenging cases (types C and D) only after the early learning phase (6 months, 25 cases), during which we only managed the easier cases (types A and B). This explains why the improvement in IOUS expertise did not show a constant trend over the three time periods, achieving the best significant results in the main outcomes only in the third period.

### Limitations

This study has some important limitations. First, the study was based on a single-institution experience, which may limit the generalizability of the findings. Additionally, the study population is not wide even though the study groups were homogenous. Another drawback is its reliance on a single surgeon, although this ensures uniformity. Extending the application of this technique to many other breast surgeons is crucial to validate the findings and explore potential variations in the learning curve, influenced by different individual experiences, approaches and attitudes. Since IOUS enables the identification of margins at risk of infiltration, a selective cavity shaving approach could be considered in a future study. We did not conduct a specific cost-benefit analysis to evaluate the cost-saving potential of IOUS. Except for the significant reduction in excised volumes, we did not conduct a comprehensive analysis of the cosmetic outcome, which should be systematically evaluated by using objective measurements and patient satisfaction questionnaires. The performances of IOUS may be limited in cases of pure DCIS and invasive lobular carcinoma, as assessing the boundaries of these lesions by US is often challenging. However, in our experience, a proper preoperative work-up helps to overcome this limitation. It is rather the unexpected DCIS component that acts as a noteworthy limiting factor, accounting for the two cases of involved margins in the IOUS group.

Finally, this study does not meet the criteria for a blind design, which may introduce a potential source of bias for the surgeon who, despite his extensive and proven experience in traditional wire- or palpation-guided surgery, has only recently introduced IOUS in his surgical practice.

## Conclusions

IOUS is the only method allowing a true real-time visualization and continuous control of resection margins during all phases of BCS. In our single-institution study, IOUS demonstrated clear superiority over TS in both oncological and surgical outcomes for all BC lesion types, with an acceptable learning curve. These findings would be further strengthened if a considerably greater number of surgeons from different BC centers achieved similar results with IOUS. Since all the other available localization techniques (including palpation) limit the surgeon’s visual guidance during BCS, IOUS could be regarded as one of the most significant modern technological innovations in the field of BC surgery, restoring sight to the breast surgeon. Studies on wider populations and dedicated randomized clinical trials are therefore needed to fully validate the technique. It is crucial to promote specialized surgical training and to incorporate IOUS in specific future guidelines.^31^ IOUS emerges as a priority skill to be developed by a modern breast surgeon, becoming a new valuable resource in any high-volume BC center and potentially a future gold standard. Our findings strongly suggest that the integration of IOUS in contemporary BCS could be regarded as a highly beneficial surgical approach. The era of blind breast surgery is hopefully coming to an end.
